# Model evaluation of target product profiles of an infant vaccine against respiratory syncytial virus (RSV) in a developed country setting

**DOI:** 10.1016/j.jvacx.2020.100055

**Published:** 2020-01-31

**Authors:** Timothy Kinyanjui, Wirichada Pan-Ngum, Sompob Saralamba, Sylvia Taylor, Lisa White, D. James Nokes

**Affiliations:** a Department of Mathematics, University of Manchester, Oxford Road, Manchester, UK; b Mathematical and Economics Modelling (MAEMOD) Research Group, Mahidol-Oxford Tropical Medicine Research Unit (MORU), Faculty of Tropical Medicine, Mahidol University, Bangkok, Thailand; c Department of Tropical Hygiene, Faculty of Tropical Medicine, Mahidol University, Bangkok, Thailand; d KEMRI-Wellcome Trust Research Programme, KEMRI Centre for Geographic Medicine Research – Coast, Kilifi, Kenya; e Nuffield Department of Medicine, University of Oxford, Oxford, UK; f School of Life Sciences and Zeeman Institute for Systems Biology an Infectious Disease Epidemiology Research (SBIDER), University of Warwick, Coventry, UK; g GSK Vaccines, Wavre, Belgium; h Peak AI, Neo, Charlotte Street, Manchester, UK

**Keywords:** RSV, Transmission model, RSV vaccination strategies, UK

## Abstract

Respiratory syncytial virus (RSV) is a major cause of lower respiratory tract disease in children worldwide and is a significant cause of hospital admissions in young children in England. No RSV vaccine has been licensed but a number are under development. In this work, we present two structurally distinct mathematical models, parameterized using RSV data from the UK, which have been used to explore the effect of introducing an RSV paediatric vaccine to the National programme. We have explored different vaccine properties, and dosing regimens combined with a range of implementation strategies for RSV control. The results suggest that vaccine properties that confer indirect protection have the greatest effect in reducing the burden of disease in children under 5 years. The findings are reinforced by the concurrence of predictions from the two models with very different epidemiological structure. The approach described has general application in evaluating vaccine target product profiles.

## 1 Introduction

Respiratory syncytial virus (RSV) was first recovered in 1955 from symptomatic chimpanzees and was known as the chimpanzee coryza agent [1]. Soon after this discovery, Chanock et al. [2], [3] isolated the virus in infants with severe lower respiratory tract infections with this work suggestive of an important role played by RSV in causing severe disease. Since then, it has increasingly been recognized that RSV represents the major viral pathogen of early childhood pneumonia worldwide with recent estimated global hospitalisations of about 3 million in children under 5 years [4]. While in absolute terms the burden of RSV is predominantly borne by developing countries, the rate of early childhood hospitalization for RSV acute lower respiratory tract infection is at least as high in industrialized countries as in the developing world, e.g. 27/1000 per year in infants 0–5 months of age (m) [5].

RSV is rapidly transmitted in communities with almost all individuals experiencing primary infections before the age of three years [6], [7]. It has also been established that RSV re-infects throughout life [8] implying that the immune response elicited by a natural infection is incomplete or temporary or both of these [9]. With increasing age, re-infections are less likely to be symptomatic in healthy older individuals [10], although can cause severe disease in the elderly [11], institutionalised individuals [12] and those with compromised immune function [13]. The infectious potential of reinfections is not fully established, though it has been shown in a household setting that the secondary attack following introduction is significantly lower for individual who are asymptomatic and shed less virus or for a shorter duration [14].

There are currently no available vaccines to prevent RSV. A monoclonal antibody, Palivizumab, is available but is high in cost compared to established infant vaccine prices, and recommended for use only in the small proportion of patients at highest risk of RSV disease i.e. prematurely born infants [15]. There is therefore need to consider other strategies to mitigate hospitalisations from RSV. There are a number of RSV vaccines under development and some in advanced stages of clinical trials [16]. Different vaccine target populations have been proposed but focus has been primarily on strategies to prevent disease in children less than 6 months of age, including infant vaccination and maternal vaccination to boost the levels, and hence duration, of transplacentally acquired antibodies against RSV in newborns [17], [18], [19]. In this piece of work we focus on the potential impact of a vaccine delivered in early infancy.

Recent work has estimated the number of hospital admissions attributable to RSV in children less than 5 years in England and Wales using time series modelling of national laboratory surveillance and hospital administrative data [20]. The study identified that of the 121,968 hospital admissions with a primary diagnosis of a respiratory tract infection between 2007 and 2012, RSV accounted for 28% (33,561). This represents a significant number of hospitalisations that could potentially benefit from an effective vaccine. We have undertaken to describe the transmission of RSV within the UK using mathematical models with an aim of simulating various infant vaccination strategies and delivery options for reducing the burden of severe disease and hospitalisations in this childhood age group. There is uncertainty as to the natural progression of RSV infection and resultant immunity to reinfection and disease, such that different model structures have previously been developed [17], [21], [22], [23], [24]. Hence, we have adopted two structurally different models developed independently which were used previously to explore vaccination strategies in a developing country setting [18].

Similarly, there remains uncertainty about the optimal characteristics of a paediatric vaccine, and the features with greatest potential for population level impact, and we have therefore explored a number of possible vaccine characteristics, referred to as target product profiles (TPPs), and report on a range of plausible scenarios. We have also discussed the potential implication of this work to future vaccine design, possible public health implication should a vaccine be introduced in the UK and potential areas for further consideration.

## 2 Methods

### 2.1 Model framework

Two age structured, deterministic, compartmental models are adopted to simulate the transmission dynamics of RSV in a developed country setting. The first model has sequential infections leading to permanent but partially immune classes, hereafter referred to as Sequential Acquisition of Immunity (SAI) model and the second assumes that partial immunity is maintained by repeated infections and wanes in its absence, hereafter referred to as Boosting and Waning of Immunity (BWI) model. The models each have a demographic sub-model, generating an appropriate age structure, and an epidemiological sub-model, describing the natural history of the infection in the population. There is also an underlying disease progression risk structure the output of which we use to fit the models to surveillance data. A vaccine delivery framework is integrated into both models incorporating the different vaccine target product profiles. For the sake of completeness, we give a short outline of the two models in the Supplementary Information but for a more rigorous account, the reader is referred to the previous study by Pan-Ngum et al. [18].

The two main differences in structure between the BWI model and the SAI model are: (a) the solid state of immunity in the SAI model (defined as P_0_, P_1_, and P_2_) is absent from from the BWI model since individuals move directly into the secondary susceptible class S_1_ after their infection. In the S_1_ class, people are less likely to get infected and if they are infected, they are at lower risk of severe infection. (b) In the BWI model, immunity can wane and secondary susceptible individuals in S_1_ can return to naïve susceptibles (S_0_) i.e. if an individual remains unchallenged for a certain length of time, they can return to S_0_ (albeit at an older age and hence altered age-related risk of disease). For simplicity, tertiary and further infections were all classified as being secondary infections in the BWI model.

#### 2.1.1 Vaccination implementation

**Fig. 1:**
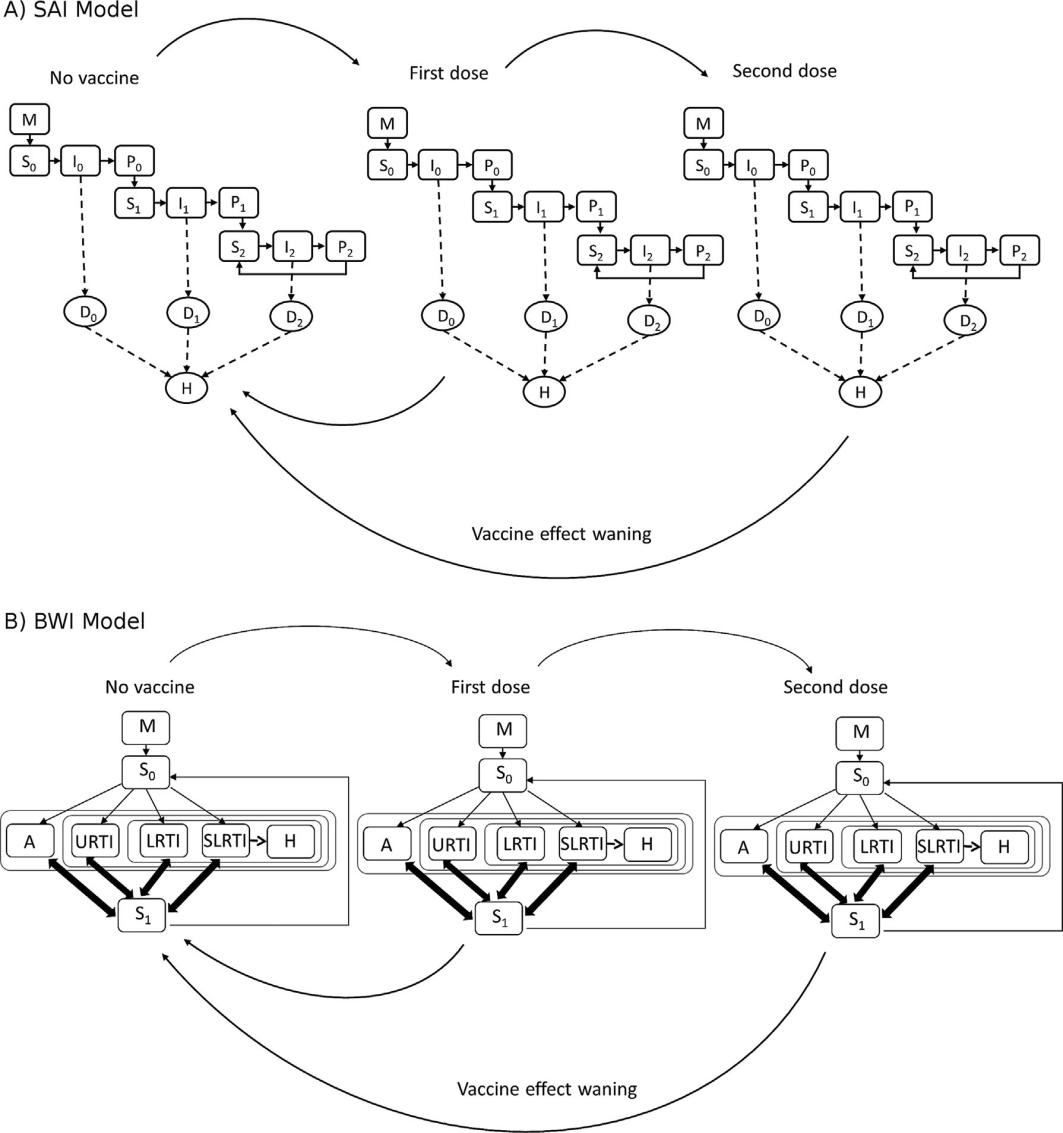
A schematic representation of the Sequential Acquisition of Immunity (SAI) and the Boosting and Waning of Immunity (BWI) model structures shown in (A) and B respectively. Vaccine effect wanes from one vaccination class into the no vaccine group.

The vaccine design and implementation allows for a vaccine that has various characteristics and can be administered in different dose regimens (with respect to number of doses and host age at delivery). The vaccine can elicit the following forms of protection: transmission blocking, reduced duration of infection, reduced infectiousness, reduced occurrence of URTI, LRTI, and severe LRTI infections. These vaccine properties are implemented in the same way in both models. The vaccine effects are acquired sequentially after a series of doses given at specific ages and wane in time from each of the doses to the non-vaccinated class. See Fig. 1.

Vaccine target product profiles

The vaccine candidates can elicit any combination of the effects shown in Table 1 from vaccinated individuals in relation to risk of infection and outcome following infection (for each a baseline choice is identified against which to make comparison of alternatives). For the dosing regimen and a description of how we model the interaction with maternal, naturally acquired and vaccine induced immunity, see the Supplementary Information

**Table 1:** Shows the vaccine effects grouped into three levels. Values in bold are the baseline TPP parameters.

**Effect**	**Low**	**Medium**	**High**
Risk of primary infection reduction	**0%**	25%	50%
Duration of infectivity reduction	0%	**50%**	75%
Infectiousness reduction	0%	**50%**	75%
Risk of URTI reduction	**0%**	50%	75%
Risk of LRTI reduction	50%	**70%**	90%
Risk of severe LRTI reduction	50%	**70%**	90%

### 2.2 Data source and model fitting

#### 2.2.1 Demographic sub-model data

The demographic sub-model for both SAI and BWI was informed by age-specific fertility and mortality rates, 2012 mid-year estimates, from the Office for National Statistics (ONS) [25]. The population distribution by age in 2012, from the same source, is used to describe the population numbers in each of the age classes. For more information, please see the Supplementary Information.

#### 2.2.2 Age specific contact data

It has become an established method to use social contact data as a proxy for contacts that are important for the spread of infections [26], [27], [28] thereby assisting in the estimation of the age-specific transmission parameters. We used the age specific physical contact rates estimates for England and Wales derived from the POLYMOD study [29]. Physical contacts have been identified as the most relevant proxy for the transmission of RSV [30]. Table S1 in the appendix gives the daily physical contact rates per age group that we have used in the models.

#### 2.2.3 England and Wales disease surveillance data

To estimate the unknown parameters in the model, we fit the model to disease surveillance data from England and Wales. We used the weekly incidence from RSV hospitalisations from Public Health England (PHE) between 2000 and 2013. The data was stratified by age from 0 to 5 years. The first 2 years was stratified by months, and the age between 3 and 5 years was stratified by yearly intervals. In the data supplied, weeks with greater than 0 and less than 5 cases were recorded as <5. For model fitting purposes, this number was transformed to 3 cases. For a description of the model fitting, please see the Supplementary Information.

To accommodate a vaccine with different characteristics from that which we have explored, we constructed a partial factorial sampling design to carry out the uncertainty analysis. The procedure takes discrete values of the parameters and considers all the possible and plausible combinations. This is referred to in the paper as the multiway sensitivity analysis (see [18] for details).

## 3 Results

**Fig. 2:**
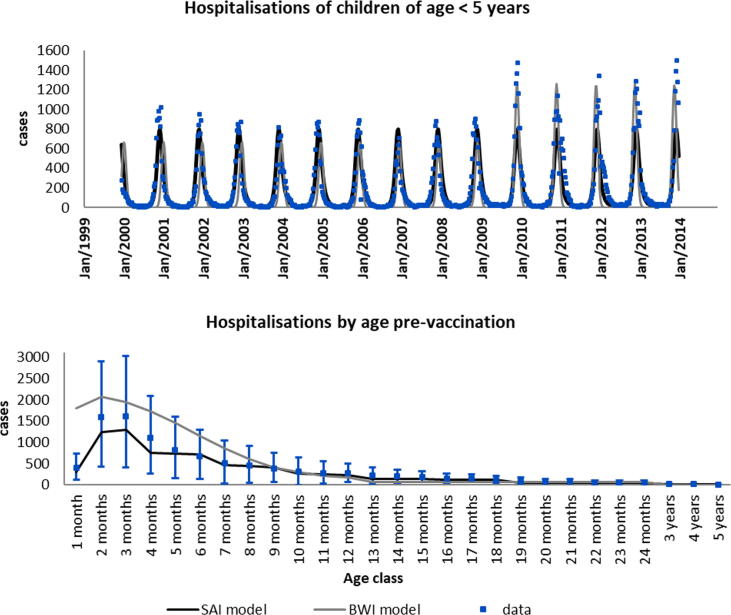
Shows the fit to the SAI (black) and BWI (grey) models to the hospitalisation data (blue) shown both by time (top) and age (bottom, with 95% CI bars). (For interpretation of the references to colour in this figure legend, the reader is referred to the web version of this article.)

Fig. 2 shows the results of fitting the two models to the RSV surveillance data. The estimated parameter values and their 95% credible intervals (CI) are given in Table 2. Both models fit the time series data well (Fig. 2A) with the BWI (grey line) capturing the increase in the peak of hospitalisations that occurred in January 2010. SAI model (black line) on the other hand generates regularly spaced epidemics with fixed amplitude. Both models however are able to capture well the timing of the start, peak and end of the epidemics. Fig. 2B shows the model fit to the same RSV specific hospitalisation data but by age. The BWI model does capture the age profile of hospitalisation well in children older than 9 months. However, it overestimates the mean number of hospitalisations in younger children between 1 month and 8 months of age. The predicted hospitalisations for these age classes are within the 95% CI except for the first month of life which is overestimated by the model. The SAI model fits the data well across all the age groups with all predictions falling within the 95% CI. Using the estimated parameters in Table 2, we proceeded to use the two models to predict the outcome of a vaccine on the number of hospitalisations over a 10 year horizon.

**Table 2:** Shows the summary of the model parameters (fixed and fitted) for both the SAI and BWI models.

**Parameter symbol**	**Description**	**SAI model**	**BWI model**	**Data source**
Seasonality parameters				
ξ	Amplitude	0.107	0.127	Fitted
ϕ	Phase angle	2.9e-07	0.902	Fitted
Transmission parameters				
q	Infectivity parameter	0.0076	0.259	Fitted
γ	Rate of recovery from primary infection, $I_{0}$ (SAI) or A, URTI (BWI) per year	$\gamma_{1}{,\gamma }_{2}=93.7$(Fixed)	$\gamma_{A},\gamma_{U}=91.25$(Fixed)	[8], [36]
	Rate of recovery from secondary and subsequent infections: $I_{1},\;I_{2}$ (for SAI) or LRTI, SLRTI (for BWI) per year	$\gamma_{0}=40.6$(Fixed)	$\gamma_{S},\gamma_{\mathrm{SL}}=40.55$(Fixed)	[8], [37]
Immunity parameters				
ω	Duration of RSV specific maternal antibody protection (in months)	1	0.242	Fitted
σ	Immunity factor reducing the susceptibility of previously exposed individuals in $S_{1}$ (both) and $S_{2}$ (SAI only)	$\sigma_{1}=0.75$(Fixed) $\sigma_{2}=0.65$(Fixed)	σ=0.528	[6]
ρ	Rate of waning of short-term immunity of recovered individuals: $P_{k}:k=0,1,2$(SAI) per year	$\rho_{0},\rho_{1},\rho_{2}=2$(Fixed)	ρ=0.277	[10], [38], [39]
α	Factor reducing infectiousness of reinfected individuals $I_{1}$ and $I_{2}$ (SAI) or SLRTI, LRTI, URTI, A (BWI)	$\alpha_{1}=0.5\alpha_{2}=0.25$(Fixed)	$\alpha_{\mathrm{SL}}=1$(Fixed) $\alpha_{L}=0.749\alpha_{U}=0.467\alpha_{A}=0.177$	See text for justification.

**Fig. 3:**
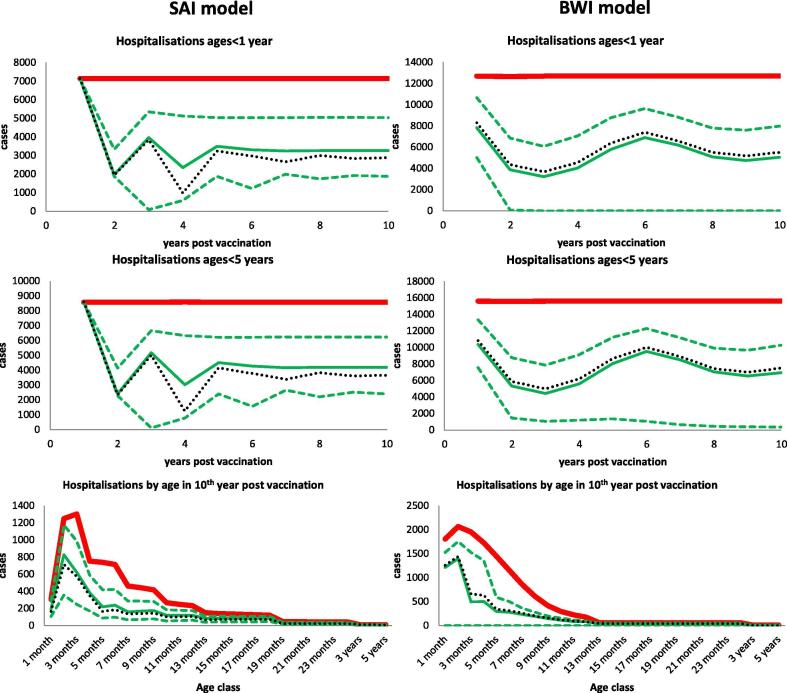
Shows the effect of vaccination. Each subplot shows the non-vaccine model prediction (solid red line), the median prediction from all TPPs (solid green line), the 95% prediction interval (dashed green lines) and the baseline TPP prediction (dotted black line). (For interpretation of the references to colour in this figure legend, the reader is referred to the web version of this article.)

Fig. 3 shows the results of implementing vaccination at time 0 for the SAI (left) and BWI (right) models with the reduction in hospitalisations in children less than 1 year of age, less than 5 years of age and the age profile 10 years after the start of vaccination shown on the top, middle and bottom rows, respectively. The figures indicate the output of 9,744 realisations of the models representing the different combinations of the vaccine TPP components with the outcome of interest being the reduction in the number of hospitalisations temporally and by age following the introduction of the vaccine. The TPP range reported only relate to the vaccine components i.e. the vaccine characteristics, vaccine dosing regimen and the duration of the vaccine. All the epidemiological parameters are held constant at their baseline values. Each graph plots the non-vaccine model prediction (solid red line), the median prediction from all TPPs (solid green line), the 95% prediction interval (dashed green lines) and the baseline TPP prediction (dotted black line). For both models, it can be noted that the median impact is very close to that of the baseline vaccine characteristics. A rapid temporal effect of vaccination is predicted by both models for age groups less than 1 and less than 5 years, with maximum (average or baseline) impact generally attained after the first two years post introduction of the vaccine. There is some predicted instability in the impact for both models, with annual fluctuations for the SAI until around year 6, and longer period oscillations for the BWI model. An equilibrium impact is approached for the SAI within the 10 year horizon but not for the BWI model. For both models, the sensitivity analysis reveals quite wide regions of possible outcome. The BWI reveals a broader range of impact than the SAI model. For the SAI model, none of the combination of vaccine characteristics lead to elimination of the hospitalisations. However, some of the BWI TPPs do result in elimination of hospitalisations. It is worth noting that none of the TPPs in both models results in the elimination of RSV infection, i.e. RSV transmission continues to occur within the population for all scenarios.

**Fig. 4:**
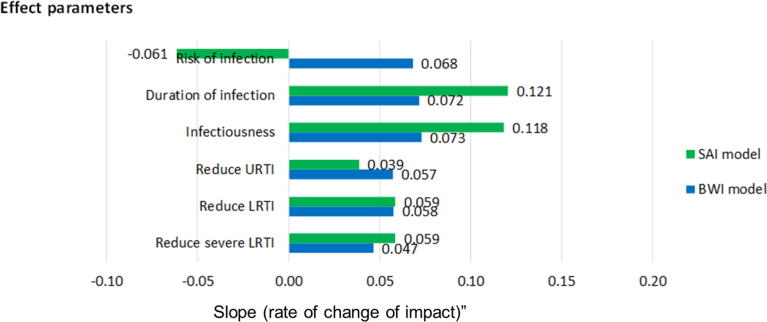
Comparison of the change in impact (at 10 years post vaccine introduction) arising from different assumptions about the vaccine characteristics (y-axis) for the SAI and BWI model shown by green and blue bars respectively. (For interpretation of the references to colour in this figure legend, the reader is referred to the web version of this article.)

An analysis of the degree of influence on the impact of specific TPP component characteristics is summarized for the two models in Fig. 4. This figure shows the multi-variate linear regression coefficients of change in impact in the x-axis (percentage reduction in under 5 year olds hospitalised) as the level of effect changes (e.g. percent reduction in risk of infection afforded by the vaccine). The outcome measure is thus the degree of influence of change of effect (of a particular type) on the outcome (the slope of the change in impact for changes in vaccine effect), and reported as the percentage change in impact for each percentage change in effect. This figure shows the relative importance, for both models, of a vaccine that reduces the infectiousness and duration of infection in reducing the number of hospitalisations in children less than 5 years old with the SAI model predicting a higher degree of reduction. There is a general agreement between the models for the other vaccine characteristics which are relatively less influential. However, the SAI model generates a counterintuitive outcome for a vaccine that reduces the risk of infection. From the figure, green bar, the model suggests, keeping other factors constant, that for each 1% increase in vaccine effect on risk of infection we expect a 0.061% increase in children under 5 who are hospitalized. This is opposed to the BWI model that predicts a 0.068% reduction in the hospitalized children for the same percentage increase in vaccine effect. We have included an explanation for this observation in the discussion section.

## 4 Discussion

We have presented an analysis of two mathematical models describing the transmission of RSV using data from the UK and explored the short term impact of an RSV vaccine with different properties and dosing schedules. The outcome of fitting the models give results that are broadly consistent but that also differ in two respects. Firstly, the BWI model tends to overestimate the burden of RSV hospitalisations in the first few months of life i.e. for children aged between 1 and 8 months. This is possibly due to the very short duration of RSV specific maternal antibodies protection estimated by the BWI model, 0.2 months compared to 1 month for the SAI model. However, it is important to note that all the model predictions do fall within the 95% credible interval and would therefore be within the range of what is expected and acceptable. Secondly, the two models are associated with slightly different seasonal temporal patterns. The BWI model is able to capture the higher peaks in number of RSV hospitalisations that occur from 2010 to 2014 which is as a result of introducing a scaling factor to the per capita rate of infection. The increase in the number of cases from 2010 was possibly a combination of “2009 pandemic-effect” and an increase adoption of multiplex PCR testing. During the 2009 pandemic, there was enhanced testing of individuals admitted to hospital with acute respiratory illness for influenza. This increased testing is likely to have been sustained afterwards with use of multiplex testing – which would also include RSV testing [31]. In contrast, the SAI model generates uniform peaks that are as a result of a sinusoidal seasonal forcing. However, both models are able to capture the peak timing and start of the annual epidemics.

Using the two models, we went on to explore the potential impact of an RSV vaccine using a range of vaccine target product profiles (TPPs). For both models, they show a consensus in the short term temporal dynamics after the start of vaccination for the baseline scenarios (i.e. for the most likely TPP). Under the baseline scenario both models predict a rapid decline in the number of hospitalisations for the first two years post vaccination and consequently achieving equilibrium at about 50% of the pre-vaccination level. Further sensitivity analysis, achieved by having different assumptions about the number of doses, vaccine characteristics and timing of doses, yields results that are in accord with the baseline programme but with a wide range of possibilities. However, the upper 95% CI of the predictions for both models always predicting a reduction in excess of 25% at the 10 year horizon, and although oscillatory in effect, the rebounds never result in increases beyond the pre-vaccination levels. In contrast, lower 95% CI for the BWI model include a reduction to zero cases hospitalised. The scenarios predicting eradication are as a result of a combination of a highly efficacious vaccine, high vaccine coverage and a long duration of vaccine protection. It is important, however, to note that although introducing vaccination in the population in both models leads to reduced transmission and consequently in hospitalisations, none of the models or TPPs leads to elimination of the infection in the population.

We have previously used the modelling framework and intervention TPPs described in this work to evaluate the effect of an RSV vaccine programme for Kenya [18]. While the models are fitted to location specific RSV infection data, there exists similarities as well as differences in the short term impact of vaccination i.e. 10 years post vaccination. In both settings, introduction of vaccination leads to a considerably rapid decline in the number of new infections in children younger than 5 years in the first 2–3 years. The vaccine characteristics that reduce the amount of virus shed and the duration of infection have the greatest benefit in reducing the burden of infection. Undoubtedly, this is because not only do they confer direct protection to the vaccinated individual but also modify the rate of infection so as to confer indirect protection to the other community members who may or may not be vaccinated. For Kenya, both SAI and BWI models predict that a transmission blocking vaccine is also quite effective in its impact for the same reasons of direct and indirect benefit. Although the BWI has the same prediction for the UK, the SAI model predicts an increase in the number of new infections in children less than 5 years for an increase in the efficacy of a vaccine that reduces the risk of infection.

At this point, we turn our attention to this counterintuitive result from Fig. 4. The SAI model predicts an increase in the number of hospitalisations with an increase the efficacy of a vaccine that reduces the risk of infection. From Fig. S1, an increase in the efficacy of a vaccine that reduces the risk of infection initially leads to a decrease in the number of hospitalisations – which is the expected result. However there seems to exist a threshold above which a more effective vaccine leads to an increase in the number of hospitalisations although importantly not beyond the pre-vaccination levels. This result is potentially influenced by the non-linear effect of the interaction between the vaccine characteristics, the duration of vaccine, the force of infection and the time at which the observations are made.

Our work suggests that immunisation of children with an RSV vaccine that reduces the infectiousness and duration of infection would have the greatest reduction in reducing the burden of severe disease requiring hospitalisations in young children. A recent study in the household setting estimated a 7 fold higher rate of RSV transmission by symptomatic children with high viral load relative to asymptomatic low viral load individuals [32]. The implication is that, a vaccine that results in reduced infectivity of individuals who are infected after vaccination (i.e. by lessening symptoms and shedding) would impact on transmission in the household, and by extension, the community, hence enhancing indirect benefits of the vaccine. These results are in accord with the predicted most beneficial characteristics of an RSV vaccine presented in this paper.

It is widely recognized that models are only as good as the assumptions behind them and the data used to parameterize them. One of the key data requirements for both models is the ‘who contacts who’ data which is a proxy measure of the potential for disease transmission given a contact between an infected and susceptible individual. For the UK setting, we used the POLYMOD data [29] which was partitioned into 5 yearly age classes. The drawback with this is that the first age class is composed of individuals who are between 0 and 5 years. And while this at first does not appear to be a concern, most of the severe RSV disease happens within the first two years of life and therefore fitting the model would require a uniform rate of contact in the first 5 years of life. This would consequently lead to either an over estimation of hospitalisations in children over two years or an underestimation of the amount of severe disease in children below 2 years of life.

Another limitation of this modelling study is the absence of hospitalisation data for individuals older than 5 years, which is a limitation across the RSV research community as there are very few hospitalisations reported in this age class. The extent to which we can make inference about the older age classes contribution to transmission and consequently disease is limited. However, if development of severe disease, at least in part, is physiological, then older individuals who have a greater residual for gaseous exchange may have a reduced risk of respiratory congestion and hence severity may decrease with increased age [6], [33]. However, if disease does not decline with age at first infection or even increases, then the model will overestimate the reduction in the amount of disease. However, we currently do not have the clinical outcome of a delayed first infection since most children will have had their primary infection in the first two years of life.

It is remarkable that for RSV, our understanding of a range of epidemiologically important features of the host-pathogen interaction remains poor. For example, (i) the nature of immunity following infection and recovery (e.g. its duration, degree of protection against infection and disease severity, and cumulative effect of reinfection); (ii) the infectivity of re-infected individuals (who tend to shed less virus, for shorter periods, and less severe symptoms, than primary infected [34]) and their role in sustaining epidemics (given that a high proportion of the population are likely infected each year [17]), and (iii) the properties of vaccines in relation to protection against infection and disease. For these reasons, in this study we adopted two structurally different model formats, and explored a wide range of vaccine properties in a sensitivity analysis. The results reveal a high degree of quantitative agreement between the two models, and regions of confidence for impact through simulations of a very large number of combinations of TPPs. We therefore hope that the work offers robust predictions in the face of such uncertainty.

Nevertheless, this modeling study has not explored all likely scenarios, for example, we assume vaccines are equally effective against both RSV groups A and B despite clear difference in cross-neutralizing antibody response [35]. Additionally, there are other vaccination strategy scenarios of public health interest, such as (i) those aimed at protecting the elderly and immunocompromised, (ii) delayed infant vaccination (where live attenuated vaccines might be more effective and better tolerated), (iii) family cocooning or school age vaccination that recognizes the significant role of elder siblings and family contacts in RSV transmission and (iv) maternal immunization to boost transplacental antibody or high potency extended half-life immunoglobulin, which might be used in combination with paediatric vaccination or household cocooning.

Future, modelling studies should explore this wider range of scenarios, incorporating additional features of importance in transmission such as explicit community structure (e.g. households [21]), and coupled to cost data and quality of life impact. These developments would yield predictions of importance to advise stakeholders both within industry who are developing vaccines and the national vaccine advisory groups such as the Joint Committee on Vaccination and Immunization in the UK.

The present paper and one published recently [18], using the same modelling approach but set in a contrasting resource and demographic setting, draw similar conclusions about optimal properties for an RSV vaccine. It will be of interest to see whether such modelling exercises have influence over vaccine development or in the selection of variables to measure during vaccine trials (e.g. infectivity upon challenge of vaccines), and whether model exploration of TPPs will in the future become a standard procedure in the vaccine development pipeline.

## CRediT authorship contribution statement

**Timothy Kinyanjui:** Conceptualization, Methodology, Software, Investigation, Writing - original draft, Writing - review & editing. **Wirichada Pan-Ngum:** Conceptualization, Methodology, Software, Investigation, Writing - original draft, Writing - review & editing. **Sompob Saralamba:** Methodology, Software. **Sylvia Taylor:** Supervision, Methodology, Writing - review & editing. **Lisa White:** Conceptualization, Methodology, Supervision, Writing - review & editing. **D. James Nokes:** Conceptualization, Methodology, Supervision, Writing - review & editing.

## Declaration of Competing Interest

The authors declare that they have no known competing financial interests or personal relationships that could have appeared to influence the work reported in this paper.
