# Supplementary material for: Model evaluation of target product profiles of an infant vaccine against respiratory syncytial virus (RSV) in a developed country setting
Source: Vaccine X. 2020 Jan 31;4:100055. doi: 10.1016/j.jvacx.2020.100055 (PMC7037978; doi:10.1016/j.jvacx.2020.100055)
Supplement: Supplementary data 1 [file mmc1.docx]

Supplementary Information

Model evaluation of target product profiles of an infant vaccine against respiratory syncytial virus (RSV) in a developed country setting

Timothy Kinyanjui^1,4,8^*, Wirichada Pan-Ngum^2,3^, Sompob Saralamba^2^, Sylvia Taylor^7^, Lisa White^2,5^, D. James Nokes^4,6^

^1^ Department of Mathematics, Alan Turing Building, University of Manchester, Oxford Road, Manchester, UK

^2^ Mathematical and Economics Modelling (MAEMOD) Research Group, Mahidol-Oxford Tropical Medicine Research Unit (MORU), Faculty of Tropical Medicine, Mahidol University, Bangkok, Thailand

^3^ Department of Tropical Hygiene, Faculty of Tropical Medicine, Mahidol University, Bangkok, Thailand

^4^ KEMRI-Wellcome Trust Research Programme, KEMRI Centre for Geographic Medicine Research – Coast, Kilifi, Kenya

^5^ Nuffield Department of Medicine, University of Oxford, Oxford, UK

^6^ School of Life Sciences and Zeeman Institute for Systems Biology an Infectious Disease Epidemiology Research (SBIDER), University of Warwick, Coventry, UK

^7^ GSK Vaccines

^8^ Peak AI, Neo, Charlotte Street, Manchester, UK

***** Correspondence: timothymuiruri.kinyanjui@manchester.ac.uk

**Keywords:** RSV; Transmission model; RSV vaccination strategies; UK

**Model framework**

Sequential Acquisition of Immunity (SAI) model

Individuals are assumed to be in any of the 10 mutually exclusive epidemiological classes. Individuals are born with temporary but solid maternal immunity (class M). They lose their protection to join the class of primary susceptibles (S_0_) from where they can become infected with a primary infection (I_0_). They then recover into the P_0_ class where they have solid, but temporary, acquired immunity. When immunity wanes following this primary infection, and similarly following subsequent infections, they move into the partial susceptible classes, S_1_ and S_2_, and then infected I_1_ and I_2_ classes, and in turn recovered P_1_ or P_2_ classes, respectively. Previously infected individuals have a reduced susceptibility to re-infection [1], shorter duration of infection [2] and reduced infectivity possibly due to shedding less virus (lower viral load or shorter duration or both [3,4]). Figure 1A shows the SAI model structure. The rates with respect to both time and age at which individuals flow from one compartment to another are described in the system of ODE described in [5].

Boosting and Waning of Immunity (BWI) model

The host population is categorized into 8 epidemiological classes, see Figure 1B, maternally protected (M), primary susceptibles (S_0_), asymptomatic (A), those with upper respiratory tract infection (URTI), lower respiratory tract infection (LRTI), severe lower respiratory tract infection (SLRTI), hospitalised (H) or secondary susceptibles (S_1_), that is, those still susceptible to infection, but have partial immunity. The proportions of people entering each class (A, URTI, LRTI, SLRTI) upon infection are both age and immune status dependent. The rates with respect to both time and age at which individuals flow from one compartment to another are described in the system of ODE described in the supplementary information (also [5].)

Dosing regimen

Infant vaccination was assumed to be either through 2 or 3 doses given at various ages.

- 2 doses at 0 and 2 m
- 2 doses at 2 and 4 m (baseline choice)
- 3 doses at 0, 1 and 2 m
- 3 doses at 2, 4 and 6 m

For the SAI model, routine vaccination is implemented as individuals pass through an age gateway e.g. for vaccination at 2m, a proportion of individuals are vaccinated as they transition out of age 1 month based on the demographic schedule. In the BWI model, a vaccination rate is applied to individuals as they pass a specified age gate such that a proportion equal to the final coverage is attained by one week in the new age class. Two options for the vaccine mean duration of effect (i.e. reciprocal of rate of waning) are considered: 1 year (baseline) and 2 years. We assume a per dose compliance of either 90% or 100 % (baseline) and this is only applied to dose 2 and 3 with final coverage levels (all doses applied) of 50%, 70% and 90% (baseline).

Interaction with maternal antibodies

We consider a number of scenarios for potential interaction of an infant vaccine in the presence of RSV specific maternal antibodies (i.e. vaccine delivery to infant in an M class). The following 3 scenarios for potential interactions are considered.

- No interaction (Baseline)

When the vaccine is administered in the presence of maternal antibodies, the vaccine has the same effect as in the absence of maternal antibodies i.e. the vaccine efficacy is not modified by the presence of maternal antibodies.


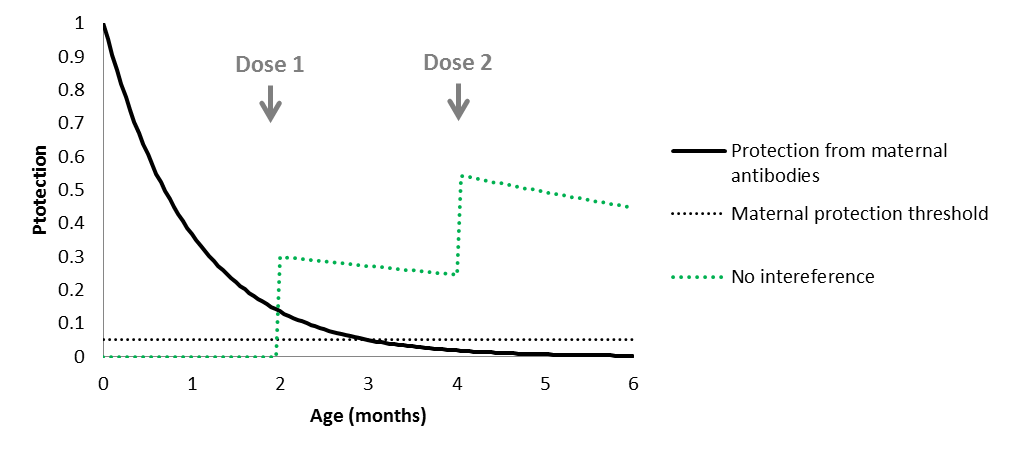


- Bounce up

If the first dose is given in the presence of maternal antibodies, then it will have no effect. If a subsequent dose is given in the absence of maternal antibodies, vaccine protection would have the same effect as on those who received the same number of doses in the absence of maternal antibodies.


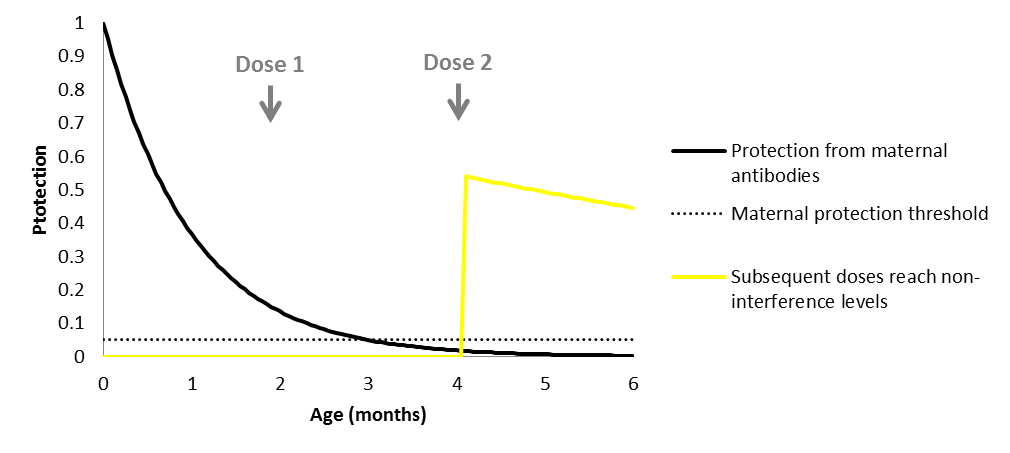


- Drop back

If the first dose is given in the presence of maternal antibodies, then it will have no effect. If the subsequent doses are given in the absence of maternal antibodies, they will have the same effect as those who receive previously doses in the absence of maternal antibodies.


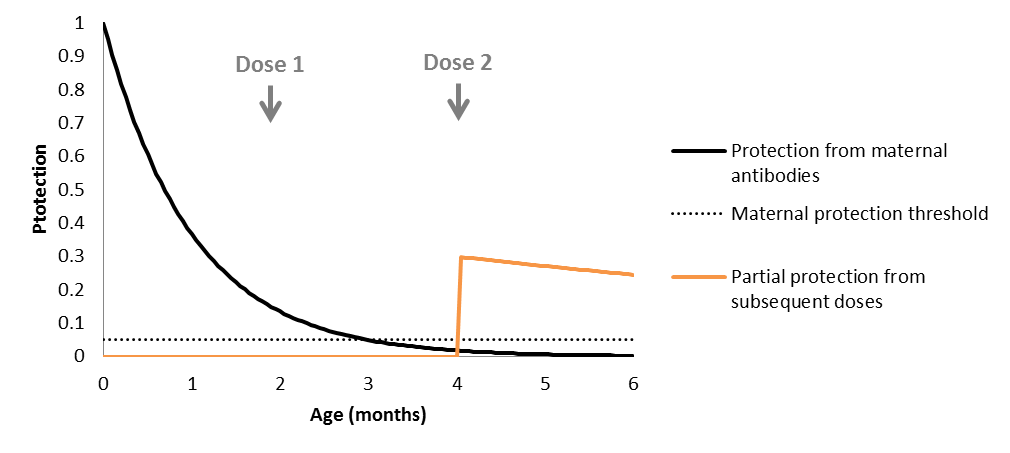


Interaction with natural and vaccine-induced immunity

A range of effects of vaccination of individuals with natural immunity are explored as listed below. Individuals with natural immunity refer to those in classes S_1_, S_2_ or those in transient protected class, P for the SAI model or S_1_ for the BWI. We considered the following three scenarios for potential interaction of a vaccine with natural or vaccine-induced immunity.

- No vaccine effect (Baseline)

If the vaccine is administered to individuals with some level of natural immunity, then it will have no effect

- Multiplicative

If the vaccine is given to individuals with some level of natural immunity, then it will have the combined reduced effect i.e. the efficacy of the vaccine will be modified by a factor that is a multiplicative function of both the natural immunity and vaccine induced immunity. For example, if V_e_ is vaccine effect and n_e_ is the natural immunity effect then the risk of infection in vaccinated individuals who already have natural immunity is calculated by (1-n_e_)λ 🡪 (1-V_e_)(1-n_e_)λ

- Top-up

When the vaccine is given to individuals with some level of natural immunity, then it would elicit protection that is equivalent to the maximum of the vaccine-induced and natural immunity. This is calculated as (1-n_e_)λ 🡪 1- max [V_e_, n_e_] λ

**Demographic sub-model**

In order to produce a rate of population growth and age structure similar to that of England and Wales in 2012, we adjusted the net rate of movement through immigration/emigration and death from all age groups by fitting the 2012 age profile data to the demographic sub-model output. This was achieved by minimizing the least squares difference between the data and the model using the set of linear equations in Eqn. 1,

$y=\sum_{a=1}^{n} \left[(A(\mu(a))^{-1}b(a))-N(a)^{*}\right]^{ 2}$ (1)

where $n$ is the number of age classes, $a$ is age, $N(a)*$is the number of people in each age class, $b(a)$ are the number of births, and $A(\mu(a))$ is the matrix of coefficients of $N$ in the ordinary differential equation (ODE) demographic sub-model. The figure below shows the demographic sub-model output (blue line) against England and Wales age specific demographic data. Yearly changes over time in birth and mortality rates are not included since this complexity would make it difficult to distinguish between the effects of vaccination driven by epidemiology and those driven by changes in the demographic pattern.


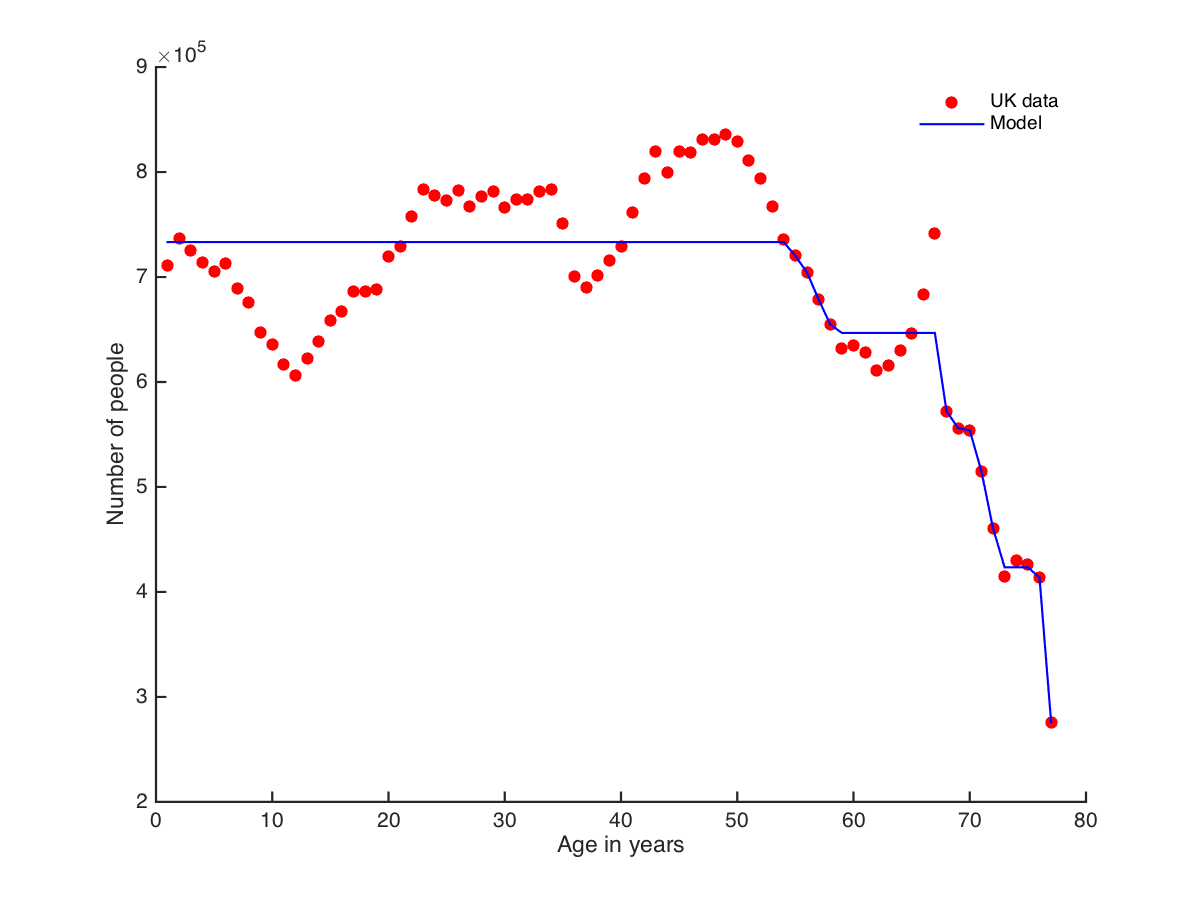


**Model fitting and uncertainty analysis**

Most of the epidemiological parameters have been identified from literature and can be found in Table 2 in the main manuscript. A parameter worth highlighting at this point is the factor reducing infectiousness of secondary and tertiary infected denoted by α1 and α2. Hall et al [6] suggested that infants shed the virus in large quantities and for prolonged periods of time. The large quantities shed can equally be attributed to primary infections since these infants are most likely experiencing their first infection. Reduction in shedding quantities is reported to be a function of age with reported decline in shedding quantities with increasing age. This means that the quantity of virus shed will also decrease with the number of previous RSV infections. We have therefore assumed that second infections are half as infectious as primary infections and third and subsequent infections are half as infectious as second infections. We have therefore estimated α1 and α2 to be 0.5 and 0.25 respectively

For the unknown parameters, denoted by ‘fitted’ in Table 2 we estimate them by fitting the model to the disease surveillance data. In fitting the models, we have used maximum likelihood estimation, which has good convergent properties, and since the disease surveillance is count data, we assume that it follows a Poisson distribution. We optimized the model parameters by maximising the log-likelihood of observing the data given the model parameters. The negative log-likelihood was used as the objective function of *fmincon* (in Matlab) and using the interior-point optimisation algorithm.

**Figures**

Figure S1: The interaction between the duration of a vaccine protection and the efficacy of a transmission blocking vaccine. Results are presented following simulations of the SAI model for a period of 10 years. Three combinations of vaccine properties and durations of vaccine protection are illustrated, grey line (52 years of vaccine protection and 80% reduction in the ability of the vaccine to reduce infectious period and amount of virus shed ), Orange line (52 years of vaccine protection and no reduction in the ability of the vaccine to reduce infectious period and amount of virus shed) and the Blue line (1 year of vaccine protection and 80% reduction in the ability of the vaccine to reduce infectious period and amount of virus shed).

**Tables**

Table S 1: Contact matrix of physical (skin to skin) contacts in Great Britain. Average number of contact persons recorded per day per survey participant.

|  |  | **Age group of participant (years)** | | | | | | | | | | | | | | |
| --- | --- | --- | --- | --- | --- | --- | --- | --- | --- | --- | --- | --- | --- | --- | --- | --- |
| **Age of contact (yr)** | | 00-04 | 05-09 | 10-14 | 15-19 | 20-24 | 25-29 | 30-34 | 35-39 | 40-44 | 45-49 | 50-54 | 55-59 | 60-64 | 65-69 | 70+ |
|  | 00-04 | 1.49 | 0.59 | 0.25 | 0.18 | 0.42 | 0.61 | 0.57 | 0.74 | 0.18 | 0.20 | 0.36 | 0.15 | 0.18 | 0.26 | 0.07 |
|  | 05-09 | 0.74 | 3.82 | 0.53 | 0.44 | 0.37 | 0.42 | 0.68 | 0.99 | 0.66 | 0.15 | 0.17 | 0.19 | 0.41 | 0.30 | 0.07 |
|  | 10-14 | 0.36 | 0.73 | 3.19 | 0.79 | 0.17 | 0.14 | 0.32 | 0.51 | 0.69 | 0.27 | 0.20 | 0.19 | 0.17 | 0.26 | 0.17 |
|  | 15-19 | 0.26 | 0.22 | 0.52 | 3.10 | 0.85 | 0.12 | 0.17 | 0.29 | 0.48 | 0.51 | 0.27 | 0.13 | 0.09 | 0.26 | 0.37 |
|  | 20-24 | 0.39 | 0.19 | 0.09 | 0.38 | 1.32 | 0.49 | 0.28 | 0.16 | 0.23 | 0.44 | 0.20 | 0.28 | 0.11 | 0.15 | 0.00 |
|  | 25-29 | 0.53 | 0.37 | 0.11 | 0.20 | 0.58 | 0.64 | 0.35 | 0.21 | 0.23 | 0.25 | 0.30 | 0.30 | 0.20 | 0.04 | 0.03 |
|  | 30-34 | 0.77 | 0.72 | 0.29 | 0.17 | 0.31 | 0.42 | 0.80 | 0.21 | 0.27 | 0.38 | 0.23 | 0.20 | 0.23 | 0.15 | 0.07 |
|  | 35-39 | 0.73 | 0.65 | 0.61 | 0.35 | 0.20 | 0.24 | 0.47 | 0.76 | 0.47 | 0.29 | 0.18 | 0.11 | 0.32 | 0.04 | 0.03 |
|  | 40-44 | 0.38 | 0.59 | 0.58 | 0.47 | 0.27 | 0.29 | 0.25 | 0.49 | 0.45 | 0.40 | 0.12 | 0.19 | 0.23 | 0.15 | 0.30 |
|  | 45-49 | 0.22 | 0.23 | 0.22 | 0.34 | 0.46 | 0.29 | 0.17 | 0.17 | 0.35 | 0.58 | 0.15 | 0.19 | 0.14 | 0.11 | 0.20 |
|  | 50-54 | 0.26 | 0.21 | 0.14 | 0.17 | 0.22 | 0.17 | 0.15 | 0.14 | 0.19 | 0.27 | 0.33 | 0.35 | 0.20 | 0.04 | 0.07 |
|  | 55-59 | 0.22 | 0.12 | 0.10 | 0.10 | 0.14 | 0.22 | 0.13 | 0.21 | 0.05 | 0.25 | 0.17 | 0.52 | 0.32 | 0.44 | 0.10 |
|  | 60-64 | 0.22 | 0.15 | 0.10 | 0.10 | 0.10 | 0.14 | 0.15 | 0.20 | 0.19 | 0.13 | 0.09 | 0.24 | 0.27 | 0.30 | 0.13 |
|  | 65-69 | 0.05 | 0.08 | 0.07 | 0.08 | 0.07 | 0.12 | 0.08 | 0.16 | 0.11 | 0.04 | 0.05 | 0.07 | 0.17 | 0.37 | 0.43 |
|  | 70+ | 0.09 | 0.09 | 0.10 | 0.08 | 0.19 | 0.10 | 0.05 | 0.13 | 0.24 | 0.29 | 0.23 | 0.22 | 0.11 | 0.22 | 0.70 |

**References**

[1] Henderson FW, Collier AM, Clyde Jr. WA, Denny FW. Respiratory-syncytial-virus infections, reinfections and immunity. A prospective, longitudinal study in young children. N Engl J Med 1979;300:530–4.

[2] Okiro EA, White LJ, Ngama M, Cane PA, Medley GF, Nokes DJ. Duration of shedding of respiratory syncytial virus in a community study of Kenyan children. BMC Infect Dis 2010;10:15.

[3] Wathuo M, Medley GF, Nokes DJ, Munywoki PK. Quantification and determinants of the amount of respiratory syncytial virus (RSV) shed using real time PCR data from a longitudinal household study. Wellcome Open Res 2017;1:27. doi:10.12688/wellcomeopenres.10284.2.

[4] Munywoki PK, Koech DC, Agoti CN, Bett A, Cane PA, Medley GF, et al. Frequent Asymptomatic Respiratory Syncytial Virus Infections during an Epidemic in a Rural Kenyan Household Cohort. J Infect Dis 2015;212:1711–8. doi:10.1093/infdis/jiv263.

[5] Pan-ngum W, Kinyanjui T, Kiti M, Taylor S, Toussaint J, Saralamba S, et al. Predicting the relative impacts of maternal and neonatal respiratory syncytial virus ( RSV ) vaccine target product profiles : A consensus modelling approach. Vaccine 2017;35:403–9. doi:10.1016/j.vaccine.2016.10.073.

[6] Hall CB, Douglas RGJ, Geiman JM. Respiratory syncytial virus infections in infants: quantitation and duration of shedding. J Pediatr 1976;89:11–5.
